# Use of Ultrasound to Diagnose Pneumonia

**DOI:** 10.5811/cpcem.2017.1.33199

**Published:** 2016-03-16

**Authors:** Derek L. Monette, Sarah E. Frasure

**Affiliations:** Brigham and Women’s Hospital, Department of Emergency Medicine, Boston, Massachusetts

## CASE REPORT

**Image 1:**
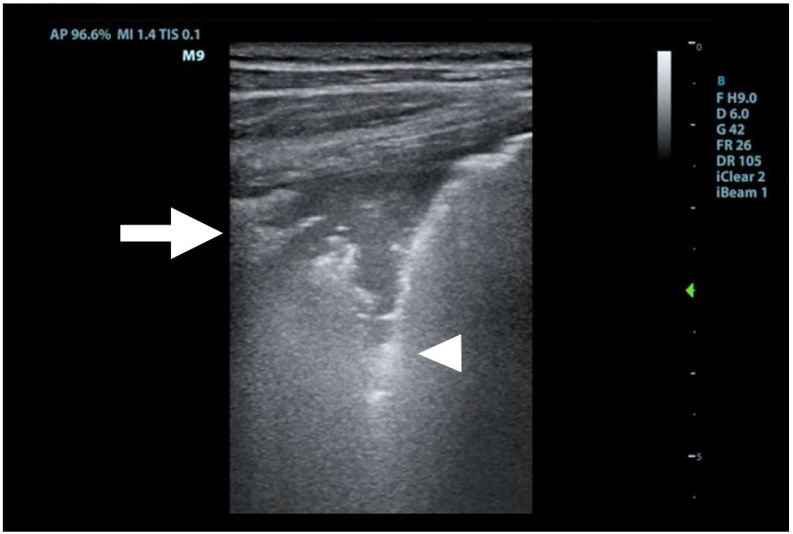
This longitudinal image of the left chest wall, performed with a linear transducer (4–12 MHz), demonstrates both a sub-pleural consolidation (arrow) and hyperechoic B-lines (arrowhead) *MHz,* Megahertz

**Image 2:**
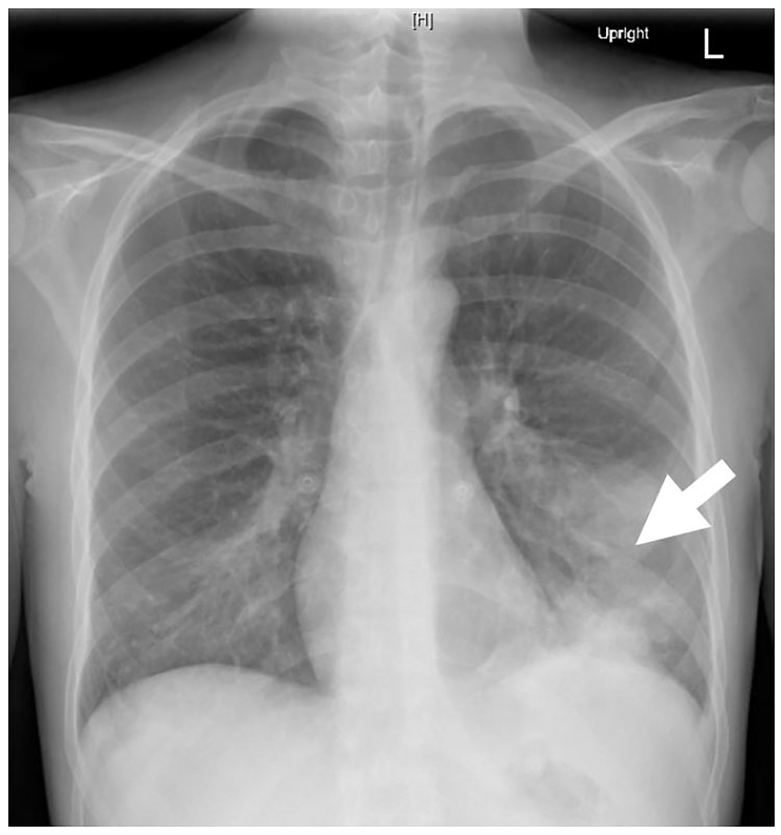
This plain film of the chest demonstrates a left lower lobe consolidation (arrow).

A 31-year-old female with a history of intravenous drug use presented to the emergency department with three days of fever, cough, and pleuritic pain. She denied orthopnea, leg swelling, chest pain, back pain, urinary frequency, sore throat, exotic travel, or recent hospitalization. Her vital signs were notable for tachycardia to 140 beats per minute. Her physical exam demonstrated left basilar crackles and a systolic murmur in the left upper sternal border. The emergency physician performed a point-of-care thoracic ultrasound (Image 1), and subsequently ordered a plain film of the chest (Image 2), which confirmed the diagnosis.

## DISCUSSION

The patient was admitted for pneumonia to the medical service and provided with intravenous antibiotics*.* The patient was admitted to the medical service and provided with intravenous antibiotics. Blood cultures grew *Streptococcus gordonii*. A transthoracic echocardiogram demonstrated the presence of a tricuspid vegetation. She was presumed to have both a community-acquired pneumonia (CAP) and long-standing endocarditis. Pneumonia remains a common cause of death in the United States and is associated with considerable morbidity and mortality, particularly in elderly and immunocompromised patients. Common causes of pneumonia include *Streptococcus pneumoniae* and respiratory viruses.^1^ A growing body of literature demonstrates the superiority of thoracic ultrasound over plain radiography in the identification of pneumonia. Classic ultrasound findings of pneumonia include air or fluid bronchograms, focal B-lines, pleural effusions, and sub-pleural consolidations.^2^ These unique findings were recognized by the emergency physician using point-of-care thoracic ultrasound, facilitating prompt diagnosis and treatment of pneumonia.

## 

Video 1 This ultrasound video clip, performed by the emergency physician, demonstrates a sub-pleural consolidation with surrounding asymmetric B-lines, consistent with pneumonia.

Video 2 This comprehensive ECHO, performed by the cardiology service during the patient’s admission, demonstrates a large tricuspid vegetation. *ECHO,* echocardiogram
